# Drug susceptibility patterns of the *Mycobacterium tuberculosis* isolated from previously treated and new cases of pulmonary tuberculosis at German-Nepal tuberculosis project laboratory, Kathmandu, Nepal

**DOI:** 10.1186/s13756-016-0129-0

**Published:** 2016-08-31

**Authors:** Gobinda Thapa, Narayan Dutt Pant, Saroj Khatiwada, Binod lekhak, Bhawana Shrestha

**Affiliations:** 1 Department of Microbiology, Goldengate International College, Battisputali, Kathmandu, Nepal; 2 Department of Microbiology, Grande International Hospital, Dhapasi, Kathmandu, Nepal; 3 Department of biochemistry, Modern Technical College, Lalitpur, Kathmandu, Nepal; 4 German-Nepal Tuberculosis Project (GENETUP), Kalimati, Kathmandu, Nepal

**Keywords:** *Mycobacterium tuberculosis*, Drug susceptibility testing, Multidrug resistant tuberculosis, Pulmonary tuberculosis, Nepal

## Abstract

**Background:**

Multidrug resistant tuberculosis (MDR-TB) is a serious public health problem in Nepal. It is a major obstacle for the control of the tuberculosis. The main objectives of this study were to determine the prevalence of the multidrug resistant pulmonary tuberculosis and to evaluate the drug susceptibility patterns of *Mycobacterium tuberculosis* isolated from previously treated and newly diagnosed cases of pulmonary tuberculosis.

**Methods:**

A cross-sectional study was conducted from March 2013 to August 2013 at German-Nepal tuberculosis project (GENETUP) laboratory, Kathmandu, Nepal. For this the sputum samples from total of 153 (49 new and 104 previously treated) suspected pulmonary tuberculosis patients were used. The diagnosis of the tuberculosis was performed by using fluorescent microscopy and culture, while the drug susceptibility testing of *Mycobacterium tuberculosis* was performed by proportion method. Lowenstein-Jensen (L-J) medium was used for the culture of *Mycobacterium tuberculosis* and the colonies grown were identified on the basis of the colony morphology, pigment production and biochemical characteristics.

**Results:**

The prevalence of MDR-TB among all the cases of culture positive pulmonary tuberculosis was 15.6 %. The rate of MDR-TB among previously treated culture positive tuberculosis patients was 19.4 % and that among newly diagnosed culture positive pulmonary tuberculosis cases was 7.1 %. The highest rate of resistance of *Mycobacterium tuberculosis*, was toward streptomycin (24.4 %) followed by isoniazid (23 %), rifampicin (17.8 %) and ethambutol (15.6 %). Among the total of MDR-TB cases among previously treated patients, highest percentage of the cases were relapse (61.1 %) followed by chronic (16.7 %).

**Conclusions:**

The high prevalence of DR/MDR-TB in our study reflects poor implementation of tuberculosis control program. On the basis of the drug susceptibility patterns of *M. tuberculosis* we found in our study, we recommend to include ethambutol instead of streptomycin in the multidrug therapy for the treatment of tuberculosis patients in Nepal. Further, due to high rate of MDR-TB among previously treated patients, we do not recommend to use first line drugs for the treatment of pulmonary tuberculosis among previously treated patients.

## Background

Tuberculosis (TB) is a communicable disease caused by *Mycobacterium tuberculosis.* Primarily, the *Mycobacterium tuberculosis* affects the lungs, causing pulmonary tuberculosis (PTB) but it may also be involved in causing extra pulmonary tuberculosis, in which sites of the body other than lungs are affected. The common mode of transmission of the tuberculosis is through droplet nuclei formed during coughing, sneezing, and speaking of the patients suffering from pulmonary tuberculosis [1].

Tuberculosis is a global public health problem with a worldwide incidence of 9 million TB cases, with 440,000 multidrug-resistant TB (MDR-TB) cases [2]. MDR-TB is defined as the TB caused by strains of *Mycobacterium tuberculosis*, which are resistant to two first-line drugs: isoniazid and rifampicin [1].

The rate of death due to tuberculosis is highest in Sub-Saharan Africa and Southeast Asia, and the majority (99 %) of the death due to tuberculosis occurs in developing countries [3]. It has been estimated that every year about 9 million new TB cases are diagnosed globally [4]. Worldwide, around 1.4 million deaths occurred in 2011 due to tuberculosis and according to World Health Organization (WHO), if proper control measures are not strengthened, by 2020 another 1 billion people will be infected with around 36 million deaths [4].

In Nepal, about 45 % of the total population is infected with *Mycobacterium tuberculosis* (both latent and active infection) [5]. The rapid spread of HIV/AIDS and the recent emergence of multidrug resistant (MDR) TB may be responsible for worsening impact of tuberculosis and increasing numbers of the tuberculosis cases [6].

The worldwide prevalence of the MDR-TB is estimated to be 5 %; with 3.5 % of the new cases and 20.5 % of the previously treated cases being MDR-TB [7]. The neighboring countries, China and India are two countries with high TB burden, accounting for around one third of the total TB cases in the world and have highest MDR-TB rates [2, 7]. Recently, there have been reports of increasing prevalence of MDR-TB [8]. The latest drug resistance surveillance of 2011 in Nepal, found the prevalence of MDR-TB to be 2.6 % and17.6 % among new and previously treated cases respectively [6]. Recently, the detection of MDR-TB in national surveillance is more dependent on GeneXpert technology [9]. The GeneXpert technology is a rapid and less labor intensive method with low biohazard risk [10]. In contrast the proportion method used in our study is time consuming, more labor intensive (requiring skilled manpower) and has high biohazard risk.

In this study, we determined the prevalence of the multidrug resistant pulmonary tuberculosis and evaluated the drug susceptibility patterns of *Mycobacterium tuberculosis* isolated from previously treated and newly diagnosed cases of pulmonary tuberculosis. This study presented the more up to date information about the prevalence of MDR-TB and drug susceptibility patterns of *Mycobacterium tuberculosis* in Nepal.

## Methods

### Study design

A cross sectional descriptive study was carried out from March 2013 to August 2013 at German-Nepal tuberculosis project (GENETUP) laboratory, Kathmandu, Nepal. For this the sputum samples from total of 153 (49 new and 104 previously treated) suspected pulmonary tuberculosis patients (having clinical and/or radiological features of tuberculosis), received from all over Nepal were used. The specimens which were culture negative and/or contaminated during culture were excluded from the study.

### Sample collection

Three early morning sputum samples (per patient) were collected in a sterile, leak proof, wide mouthed, transparent plastic container. Saliva and nasal secretions were not accepted.

### Microscopy

For microscopy auramine-phenol fluorochrome staining was used [11].

### Sputum culture for *Mycobacterium tuberculosis*

Method devised by Kent and Kubica was used for the digestion, decontamination and concentration of sputum samples [12]. For culture L-J medium was used. The colonies grown were identified on the basis of the colony morphology, acid fast staining, pigment production and biochemical characteristics [13].

### Drug susceptibility testing by proportion method

Drug susceptibility testing of *Mycobacterium tuberculosis* was performed by proportion method as suggested by Heifets [14].

### Quality control

For quality control, *M. tuberculosis* H37Rv was inoculated into a series of drug containing media and control media and incubated along with media inoculated with the test strains.

### Statistical analysis

Data were analyzed using statistical package for the social sciences (SPSS) version 16.0. The *χ*^2^ test was used and *p*-value < 0.05 was considered statistically significant.

## Results

Among the total of 153 patients, sputum samples from 129 patients were smear positive and those from 135 patients were culture positive. Similarly, sputum samples from 116 patients were both culture and smear positive. Further, sputum samples from 19 patients were smear negative but culture positive. In addition, samples from five patients were both culture and smear negative. Sputum samples from 13 patients were contaminated during culture.

### Case-wise distribution of PTB patients

Out of 135 *Mycobacterium tuberculosis* isolates, 31.1 % (*n* = 42) isolates were isolated from new TB cases and 68.9 % (*n* = 93) isolates were isolated from the previously treated TB cases. Among previously treated TB cases the highest number of cases were relapse (59.1 %) followed by treatment failure cases (15.1 %), chronic cases (11.8 %), return after default cases (8.6 %) and follow-up cases (5.4 %).

### Drug susceptibility patterns of *Mycobacterium tuberculosis*

Of total 135 *Mycobacterium tuberculosis* isolates, 68.9 % (*n* = 93) were sensitive to all four drugs and 31.1 % (*n* = 42) were resistant to any drugs. The highest rate of resistance was toward streptomycin (SM or S) (24.4 %) followed by isoniazid (INH or H) (23 %), rifampicin (RMP or R) (17.8 %) and ethambutol (EMB or E) (15.6 %). Among the total of 135 culture positive TB cases, 21(15.6 %) were found to be MDR-TB. The prevalence of MDR-TB (19.4 %) among the previously treated cases was significantly higher than that among newly diagnosed cases (7.1 %) and the difference was statistically significant. Around 6.7 % of the total culture positive TB cases were poly-resistant TB (resistant to more than one drug but not MDR-TB). No poly-resistant TB cases were reported among newly diagnosed tuberculosis patients (Table 1).

**Table 1:** Drug susceptibility patterns of *Mycobacterium tuberculosis* isolated from new and previously treated cases of pulmonary tuberculosis

Susceptibility patterns	Total cases (*N* = 135) n (%)	New cases (*N* = 42) n (%)	Previously treated cases (*N* = 93) n (%)
Any drug resistance	42 (31.1)	9 (21.4)	33 (35.5)
Any isoniazid resistance	31 (23)	3 (7.1)	28 (30.1)
Any rifampicin resistance	24 (17.8)	4 (9.5)	20 (21.5)
Mono isoniazid resistance	2 (1.5)	0	2 (2.2)
Mono rifampicin resistance	2 (1.5)	1 (2.4)	1 (1.1)
MDR-TB	21 (15.6)	3 (7.1)	18 (19.4)
Poly-resistant TB (resistant to more than one drug, but not MDR-TB)	9 (6.7)	0	9 (9.7)

### Drug susceptibility patterns of *Mycobacterium tuberculosis* isolated from different cases of previous treated patients

Among the total of MDR-TB cases among previously treated patients, highest percentage of the cases were relapse (61.1 %) followed by chronic cases (16.7 %), treatment failure cases (11.1 %), return after default cases (5.6 %) and follow-up cases (5.6 %). Drug susceptibility patterns of *Mycobacterium tuberculosis* isolated from different groups of previously treated patients are presented in Table 2.

**Table 2:** Drug susceptibility patterns of *Mycobacterium tuberculosis* isolated from different groups of previously treated patients

Susceptibility patterns	Total cases (*N* = 93) n (%)	Relapse (*N* = 55) n (%)	Treatment failure (*N* = 14) n (%)	Return after default (*N* = 8) n (%)	Follow up (*N* = 5) n (%)	Chronic (*N* = 11) n (%)
Any drug resistance	33 (35.5)	18 (32.7)	4 (28.6)	4 (50)	1 (20)	6 (54.6)
Any isoniazid resistance	28 (30.1)	16 (29.1)	2 (14.3)	3 (3.8)	1 (20)	6 (54.6)
Any rifampicin resistance	20 (21.5)	11 (20)	3 (21.4)	2 (25)	1 (20)	3 (27.3)
Mono isoniazid resistance	2 (2.2)	1 (1.8)	0	0	0	1 (9.1)
Mono rifampicin resistance	1 (1.1)	0	1 (7.1)	0	0	0
MDR-TB	18 (19.4)	11 (20)	2 (14.3)	1 (12.5)	1 (20)	3 (27.3)
Poly-resistant TB (resistant to more than one drug, but not MDR-TB)	9 (9.7)	4 (7.3)	0	4 (50)	0	2 (18.2)

Note: Relapse cases are the patients who become symptomatic after completion of the treatment, treatment failure cases are the patients who remain smear positive or culture positive even after 5 or more months of start of anti-tubercular therapy, default cases are the patients whose treatment is halted for at least two consecutive months, follow up cases are the patients who are called in follow up even after successful completion of anti-tubercular therapy, chronic cases are the patients who have received repeated incomplete treatment and who still have active tuberculosis that is considered to be incurable [6]

## Discussion

Similar to our finding, Hajoj et al. in Saudi Arabia reported the prevalence of MDR-TB among previously treated TB patients to be 15.9 %, however they showed lower rate of MDR-TB (in comparison to our study) among newly diagnosed cases (1.8 %) [15]. Similarly, Rijal et al. in their study during 2003 to 2004 found the rates of MDR-TB among previous treated and new cases to be 19.3  and 2.6 % respectively [16]. However, Subba et al. reported quite high rate of MDR-TB cases (i.e. 22.2 % among new cases and 37.2 % among previously treated cases) in a study during 2005 to 2006 [17]. Further, during 2006 to 2007 Pradhan et al. discovered the prevalence of MDR-TB to be 15.5 % among previously treated cases and 4.2 % among new cases [18]. The most recent drug resistance surveillance of 2011 in Nepal, reported the prevalence of MDR-TB to be 2.6 and 17.6 % among new and previously treated cases respectively [6]. Another study during 2011 to 2012 by Tharu et al. noted the rate of MDR-TB among culture positive retreatment cases to be 33 % [19]. A study conducted by Khunjeli et al. in Nepal from 2012 to 2014 discovered the prevalence of primary drug resistant tuberculosis to be 4.8 % [20]. The drug susceptibility patterns of *M. tuberculosis* isolated in different studies conducted in Nepal during different periods of time have been presented in Table 3 [16–18, 21]. Over the years, there has been gradual increase in primary MDR-TB in Nepal. This may be attributed to the increased rate of the acquired resistance, which contributes to the rise in primary drug resistant TB [6]. The main causes of high rates of drug resistance among previously treated tuberculosis cases in Nepal may be improper and/or incomplete treatment [22]. Other risk factors for development of MDR-TB are previous TB treatment, smoking habit, poverty, illiteracy, migration to/from high MDR-TB prevalence areas, alcoholism, poor housing, overcrowding and homelessness [23, 24]. The mode of transmission of drug-resistant TB is same as that of drug susceptible TB. And the transmission of the MDR-TB is a very serious public health issue, as it is very difficult to treat MDR-TB.

**Table 3:** The drug susceptibility patterns of *M. tuberculosis* isolated in different studies conducted in Nepal during different periods of time

	National survey 1996–1997 [21]		National survey 1998–1999 [21]		National survey 2001–2002 [21]		Rijal et al. 2003–2004 [16]		Subba et al. 2005–2006 [17]		Pradhan et al. 2006–2007 [18]		National survey 2006–2007 [21]		Finding of our study 2013	
	New cases	Previously treated cases	New cases	Previously treated cases	New cases	Previously treated cases	New cases	Previously treated cases	New cases	Previously treated cases	New cases	Previously treated cases	New cases	Previously treated cases	New cases	Previously treated cases
Any drug resistance	9.8 %	0	13.2 %	28.6 %	11.0 %	40.9 %	36.8 %	75.2 %	64.5 %	74.8 %	46.5 %	67.2 %	4.7 %	25.3 %	21.4 %	35.5 %
Mono-drug resistance	5.7 %	0	7.6 %	11.6 %	7.0 %	13.0 %	23.7 %	32.3 %	20 %	23.6 %	24.3 %	25.9 %	9.1 %	6.1 %	14.3 %	6.5 %
Multidrug resistance	1.1 %	0	3.7 %	12.5 %	1.32 %	20.5 %	2.6 %	19.3 %	22.2 %	37.2 %	4.2 %	15.5 %	2.9 %	11.7 %	7.1 %	19.4 %
Resistance to all 4 first line drugs	0	0	1.8 %	9.8 %	0.8 %	9.4 %	2.6 %	11.8 %	6.7 %	18.8 %	0	6.9 %	1.8 %	6.8 %	4.8 %	15.1 %

In accordance to our finding, in a study by Massi et al., 60.9 % of the total *M. tuberculosis* isolates were found to be sensitive to all four drugs, while 39.1 % of the isolates were resistant to at least one of the four drugs [25]. However, Pradhan et al. [18] and Rijal et al. [16] noticed the rates of any drug resistance to be 52.5 and 67.8 % respectively.

Higher rate of sensitivity to RMP alone is a good indicator for success of directly observed treatment short course (DOTS) program but in our study 9.5 % of mono-resistance to RMP was observed in new patients showing the less success of the DOTS program in Nepal. Similarly, in new cases mono-resistance to INH was 7.1 %. Pradhan et al. [18] showed the mono-resistance among new cases to RMP and INH to be 4.2 and 1.4 % respectively. Similarly, in a study by Rijal et al. [16] mono-resistance among new cases to RMP and INH were found to be 0 and 5.3 % respectively. Subba et al. [17] also reported the mono-resistance among new cases to RMP and INH to be 0 and 13.33 % respectively. Green et al., reported 6.1 % resistance to RMP and 17.7 % resistance to INH from South Africa [26]. The resistance of *Mycobacterium tuberculosis* to RMP and INH is an alarming marker of MDR-TB [27]. As in our study the mono-resistance to streptomycin was reported to be highest in the studies by Pradhan et al. [18] and Rijal et al. [16]. This may be explained by the extensive use of streptomycin for the treatment of other bacterial infections. The use of standard anti-tubercular therapy in mono-DR-TB and poly-DR-TB posses a great risk of treatment failure leading to the development of MDR-TB in case of the mono-drug and poly-drugs (non-MDR) resistant cases [26]. The differences in the susceptibility patterns of *M. tuberculosis* in other countries and in Nepal, may be due to the difference in effectiveness of tuberculosis control program in different countries and difference in other risk factors like previous TB treatment, improper and/or incomplete treatment, smoking habit, poverty, illiteracy, migration to/from high MDR-TB prevalence areas, alcoholism, poor housing, overcrowding and homelessness in different countries. Further, difference in the frequently used antibiotics from one country to another may also contribute to difference in drug susceptibility patterns among different countries.

Findings of our study suggest that relapse cases are predominant over the other previously treated tuberculosis cases. Similar to our study, Al-Marri (85 %) in the state of Qatar [28] and Subba et al. (68.4 %) in Nepal [17] reported that the highest number of cases in previously treated TB patients were relapse.

The presence of DR/MDR-TB reflects poor tuberculosis control in the present or past which creates great problem to effective implementation of TB control program throughout the world [15]. So, for the effective implementation of the TB control program, it is necessary to have the laboratories where MDR-TB cases can be detected but they are not sufficiently available in developing countries like Nepal [6]. In case of under developing countries like Nepal, the rapid GeneXpert MTB/RIF assay may be proved to be promising for detection of MDR-TB cases. Early diagnosis, good quality treatment under DOTS program, early screening and treatment in closed contact and infection control program may be very useful to improve health care service, hence to control the MDR-TB.

DOTS can treat virtually all the patients with drug susceptible TB [29]. And for the patients with MDR-TB, DOTS Plus can be used for the treatment and to prevent the development of the further drug resistance [30]. But under DOTS Plus second-line injectable drugs, which are inherently more toxic and less effective than first-line drugs, are used daily for 2 years [30]. The treatment is more expensive and prolonged. So it is necessary to confirm MDR-TB (before starting treatment) in case of failure of DOTS rather than presuming the patients failing DOTS to have MDR-TB and starting the 2nd line anti-tubercular therapy [30].

### Limitations of the study

Due to lack of resources, we could not include more samples in our study. Multi-center studies including large number of samples could have generated more significant data. We could not use molecular methods for the identification of the *Mycobacterium tuberculosis*.

## Conclusions

In our study high rate of multidrug resistance was noted. On the basis of the drug susceptibility patterns of *M. tuberculosis* we found in our study, we recommend to include ethambutol instead of streptomycin in the multidrug therapy for the treatment of tuberculosis patients in Nepal. Further, due to high rate of MDR-TB among previously treated patients, we do not recommend to use first line drugs for the treatment of pulmonary tuberculosis among previously treated patients. The empiric treatment of tuberculosis should be based on the local drug susceptibility patterns of *M. tuberculosis*. Rapid testing for drug resistance by some methods like GeneXpert MTB/RIF assay and improvement of infection control program may be helpful in control of the tuberculosis.

## Abbreviations

AIDS, acquired immune deficiency syndrome; DOTS, directly observed treatment short course; DR-TB, drug resistant tuberculosis; DST, drug susceptibility testing; EMB or E, ethambutol; GENETUP, German-Nepal tuberculosis project; HIV, human immunodeficiency virus; INH or H, isoniazid; L-J, Lowenstein-Jensen medium; MDR-TB, multidrug-resistant tuberculosis; PTB, pulmonary tuberculosis; RMP or R, rifampicin; SM or S, streptomycin; SPSS, statistical package for the social sciences; TB, tuberculosis; WHO, world health organisation
